# Selective Pressure along a Latitudinal Gradient Affects Subindividual Variation in Plants

**DOI:** 10.1371/journal.pone.0074356

**Published:** 2013-09-19

**Authors:** Mar Sobral, José Guitián, Pablo Guitián, Asier R. Larrinaga

**Affiliations:** 1 Department of Biology, Stanford University, Stanford, California, United States of America; 2 Department of Cellular Biology and Ecology. University of Santiago de Compostela, Santiago de Compostela, Spain; 3 Department of Botany, University of Santiago de Compostela, Santiago de Compostela, Spain; 4 Mediterranean Institute for Advanced Studies, Esporles, Spain; CNRS, Université de Bourgogne, France

## Abstract

Individual plants produce repeated structures such as leaves, flowers or fruits, which, although belonging to the same genotype, are not phenotypically identical. Such subindividual variation reflects the potential of individual genotypes to vary with micro-environmental conditions. Furthermore, variation in organ traits imposes costs to foraging animals such as time, energy and increased predation risk. Therefore, animals that interact with plants may respond to this variation and affect plant fitness. Thus, phenotypic variation within an individual plant could be, in part, an adaptive trait. Here we investigated this idea and we found that subindividual variation of fruit size of *Crataegus monogyna*, in different populations throughout the latitudinal gradient in Europe, was explained at some extent by the selective pressures exerted by seed-dispersing birds. These findings support the hypothesis that within-individual variation in plants is an adaptive trait selected by interacting animals which may have important implications for plant evolution.

## Introduction

Biologically meaningful variation in nature ranges from the level of individuals up to the scale of biomes. Variation among individuals provides the raw material for natural selection to operate leading to evolutionary change. Therefore individuals are traditionally considered the lowest level at which evolutionary or ecologically meaningful variation occurs. However, natural selection acts on phenotypes and a single individual (genotype) can produce a set of different phenotypes depending on the environmental conditions, a process known as phenotypic plasticity. Modular organisms, such as plants, produce a multiplicity of repeated structures like leaves, flowers and fruits which are phenotypically different and can be considered “re-runs” of the same genotype under different internal and external micro-environmental conditions [1].

Subindividual variation, the phenotypic variation among repeated organs within the same individual, is caused by a complex web of factors including ontogenetic contingency, organ-level reaction norms and developmental instability. Ontogenetic contingency refers to the combined effect of location within the plants, previous developmental history and localized environmental characteristics [2], [3], [4]. Organ level reaction norms are functions that link micro-environmental variation occurring within an individual to variation in the expression of phenotype [1]. Developmental instability is the proportion of phenotypic variance in organ traits that remains unexplained after accounting for the organ’s reaction norm [4]. One component of variation within individuals that may have a genetic basis and could be affected by natural selection is organ-level reaction norms, which cause organ-level phenotypic plasticity [1]. We explore here the potential evolutionary implications of subindividual variation in plant phenotypic traits.

Subindividual variation in plants is evolutionarily significant only if individuals differ in their levels and/or patterns of variation, if such variation has a genetic basis, and if it affects plant fitness. First, despite that the ecologically and evolutionary implications of variation within-individual plants have been generally disregarded, some studies have shown its genetic basis in wild plants. For example, a genetic basis for subindividual variation has been demonstrated for several flower traits [5], [6], [7], leaf traits [5], [8] and seed traits [9]. A higher quantity of studies have shown a genetic basis for phenotypic variation within individual plants in cultivated species; as for example, it has been shown for fruit traits [10]. Second, interacting animals could respond to the levels of variation within-individual plants, affecting plant fitness [1], [11]. This preference could be due to the ecological costs imposed by such variation on interacting animals, for example, time and energy costs, increased predation risk and constraints on optimal foraging [1]. Constraints on optimal foraging refer to the effect that variability in reward has over the overall perceived quality of an individual plant. For example, foraging preferences of frugivorous birds responded to variation in reward [12]. Besides the genetic basis of within-individual variation and its relationship with animal behaviour and individual fitness, stronger evidence of its adaptive value would be provided if different populations of a plant species varied in the levels of subindividual variation in some organ trait and these differences were explained by the selective pressures exerted by the interacting animals within particular populations [1].

Here, we tested the hypothesis that variation within-individual plants in fruit size of *Crataegus monogyna* (hawthorn) is, at some extent, shaped by selective pressures exerted by seed-dispersing birds. In order to do that, we intended to find out if differences in fruit size subindividual variation among populations of hawthorn, throughout its latitudinal range of distribution in Europe, were explained by the selective pressures exerted by the seed-dispersing birds within each population. It has been shown that hawthorn seed dispersers have innate fruit size preferences in aviary conditions [13] and respond to different degrees of fruit size variation within plants exerting phenotypic selection on fruit size variation in natural settings [11]. Variation in fruit size of hawthorn could be selected in specific situations. For example, we could expect the selection of plant individuals with lower variation in fruit size, since it may reduce assessing time and predation risk [1]. However, a situation in which trees compete for dispersers, which could vary in fruit size preference, may imply that trees with a higher variability in fruit size may have a higher fitness. The coexistence of small and large fruit sizes may be positive for the tree in these situations. Therefore, the composition of the avian disperser guild and the relative abundance of each species will influence the kind of phenotypic selection exerted in each particular population. The different species dispersing hawthorn seeds vary in morphological and behavioral characteristics such as body size, treatment of seeds in the digestive tract, migrating behaviour and habitat use. Additionally, the relative role that bird species represent in the disperser guild varies among populations throughout the latitudinal gradient of distribution of the species [14]. Consequently, hawthorn seed dispersers may exert different selective pressures among populations. Therefore, *C. monogyna* and its seed-dispersing birds provide an optimal system for studying the adaptive value of subindividual variation.

To study the effect that selective pressures exerted by seed-dispersing birds may have on the variation of fruit size in hawthorn, we examined the differences in within-individual variation among populations, across the latitudinal range of distribution in Europe, and we determined the factors affecting them, teasing apart the effect of selective pressures exerted by the seed-dispersing birds from other factors. We found that selective pressures exerted by seed-dispersing birds affected the differences in subindividual fruit size variation among populations. These findings are consistent with the hypothesis that, variation is a trait subject to natural selection exerted by interacting animals [1].

## Methods

Hawthorn (*Crataegus monogyna*, Rosaceae) is a shrub or small tree with fleshy fruits that contain a single seed. It is distributed over most of Europe, northern Africa and western Asia, and has been introduced into North America [15]. In Europe, hawthorn fruits are mostly consumed by *Turdus merula* (blackbird), *Turdus iliacus* (redwing), and *Turdus philomelos* (song thrush) [14]. These birds defecate the seeds away from the mother plant, giving the seeds the chance to escape from resource competition and negative density-dependent effects such as pathogen infection or seed predation [16]. In fact, almost all non-dispersed seeds are preyed upon by mice, and germination under adult plants is virtually absent (personal observation). Therefore feeding preferences of birds could impact the fitness of hawthorn individuals. Birds swallow fruits in a longitudinal fashion this is why the size constraints imposed by gape width are determined by fruit diameter rather than length [17]. Additionally, the diameter and length of fruits and seeds are under different phenotypic selective pressures. Selection exerted by seed-dispersing birds is different for seed length and diameter. The targets of selection by birds in this species are fruit diameter and seed length [11]. For this reason, in the current study fruit size refers to fruit diameter and seed size refers to seed length.

We sought to separate the effect of seed-dispersing bird’s selection from other effects that could affect the expression of subindividual variation in fruit size. We assessed the effect of the selective pressures exerted by birds in different populations after taking into account the effects of precipitation, latitude and plant correlated traits (i.e., crop size and seed size). Latitude is related to some abiotic factors as radiation and temperature as well as to community composition. Hence, by including latitude we conflated a broad set of abiotic and biotic sources of variation others than those explicitly considered in the analysis.

**Figure 1:**
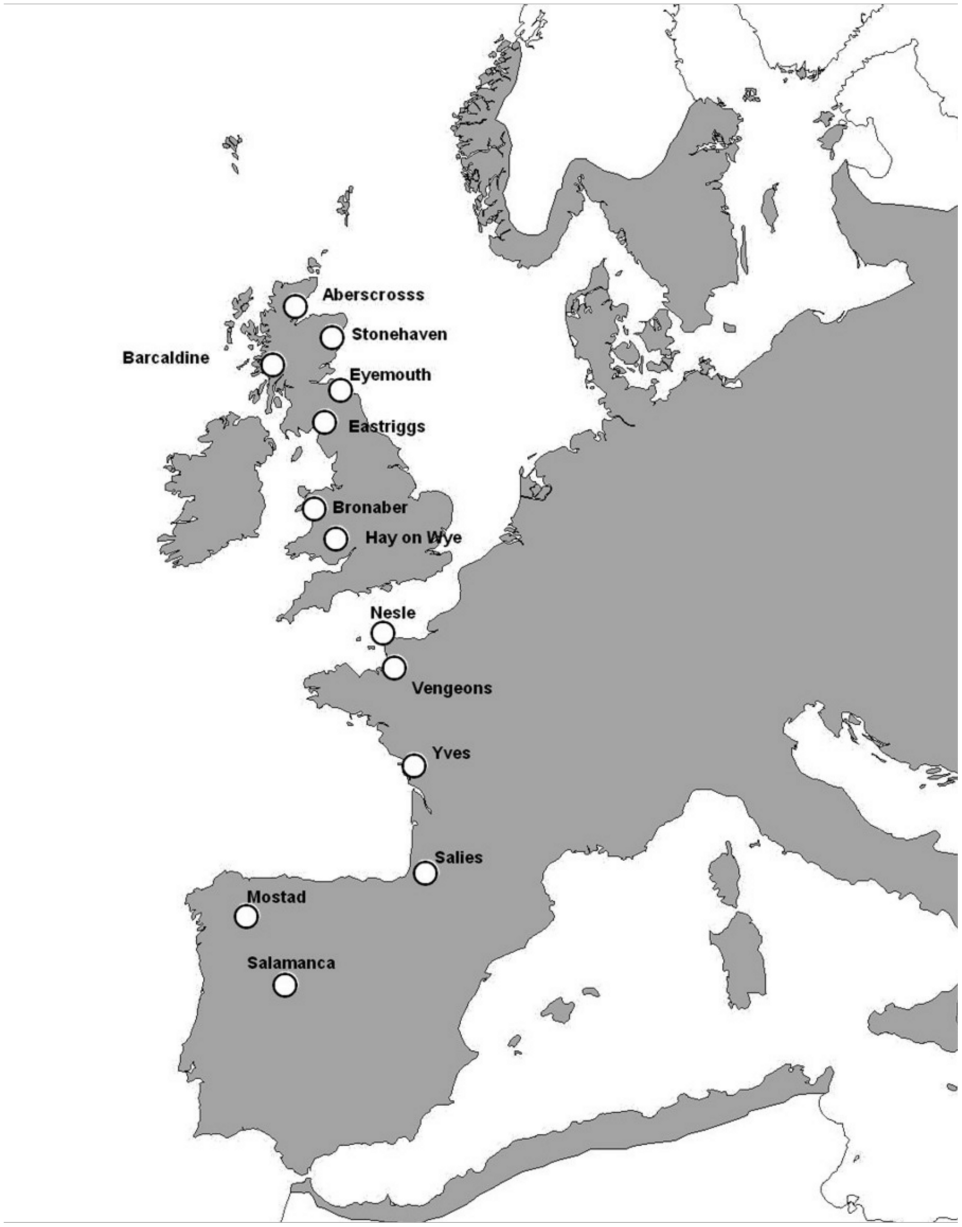
Location of the *Crataegus monogyna* populations studied. The limits of the natural distribution of the plants in Europe are shown in grey [15].

In 2007, we studied 13 populations of *C. monogyna* throughout the latitudinal range of the species in Europe (Figure 1). No specific permissions were required, when in private land, owners were asked permission to enter the sites and conduct the study. The study did not involve any endangered or protected species. We obtained precipitation data on the 13 populations studied from the National Oceanic and Atmospheric Administration USA *(*NOAA) (http://www.cdc.noaa.gov:80/USclimate/). From these data we assessed the cumulative precipitation between April and September 2007. During these months fruits are formed and ripen, so the availability of water during this period could affect fruit and seed size. When fruits were still on the trees and had not yet been eaten by the avian dispersers (October 1–19, 2007), we selected and marked 25 trees in each population. We measured the area under the canopy and estimated crop size. In each tree we haphazardly marked five branches and counted their fruits. We marked three 0.5×0.5 m areas under each tree to estimate the number of fallen fruits for the duration of the experiment. We collected a sample of 25 fruits from each of the trees to measure the average fruit and seed size and their subindividual variation. We had previously tested that average and variation in fruit diameter and seed length were not different among random samples of 25 fruits and 100 fruits of the same tree. We measured the length and diameter of the fruits and seeds collected from the 325 trees (8,125 fruits and seeds), with a 0.01 mm precision caliper. On a second visit to each population (December 1–18, 2007), after the birds had consumed most of the fruits and when the fruits left in the trees were already rotten, we counted the number of fruits remaining on the marked branches and the number of fruits found in the marked areas under the canopies. During the period between both visits some of the trees were cut down, and hence the final number of trees used in the phenotypic selection analysis was 271.

### Statistical Analyses

- Selective pressures exerted by seed-dispersing birds

Phenotypic selective pressures were assessed following the methods of [11]. We estimated the number of dispersed seeds per tree (D) as:
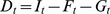
where *I_t_* is the initial crop of tree *t*, *F_t_* the final standing crop of tree *t* and *G_t_* the number of fallen fruits under tree *t*. *G_t_* was estimated as:



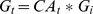
where *G_i_* stands for the average fallen fruit density across the quadrats during the study period, and *CA_t_* for the projected area of the canopy. The number of dispersed seeds per tree (*D_t_*) was used as the fitness component in the phenotypic selection analysis. By considering dispersal rates as the response variable from which relative fitness is to be calculated, we assume that dispersed seeds have higher fitness than undispersed seeds, due to reduced competition (especially mother and half-sibling competition), reduced density-dependent effects (pathogens, parasites, post-dispersal seed predators) and the colonization of suitable microsites [16]. Finally, seeds which are dispersed receive a gut treatment which helps them to germinate [18]. Additionally, seed predation under adult plants is near 100% in the populations we studied (personal observation), nor did we observe germination under adult plants. In long-lived organisms measuring total fitness is often not possible; hence we used a measure of reproductive success as a proxy for fitness, a common approach in the phenotypic selection literature [19].

We quantified the subindividual variation in fruit and seed traits by means of the Coefficient of Variation (CV) of these traits, since variance and standard deviation are scale-dependent and therefore cannot be used to compare variation levels [20], [21]. Total selection was assessed as the selection differential (S), that is, the standardized coefficient of a simple regression of relative fitness on each trait [22], [23]. We used the ordinary least squares regression to estimate the selection coefficients without transforming fitness to achieve normality [22]. By including variation (CV) as a separate trait we adhere to the variance-aware extended model of Herrera [1], which takes into account not only the average value of the traits, but also their subindividual variation.

The relative fitness of tree *t* was defined as the proportion of dispersed seeds of tree *t* relative to the mean number of dispersed seeds per tree of the population:
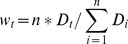
where *n* is the number of trees in each population, *D_t_* is the number of dispersed seeds of tree *t* and *D_i_* the number of dispersed seeds of tree *i*.

- Phenotypic differences among populations. Effect of the selective pressures exerted by seed-dispersing birds

In order to analyze how fruit and seed size variance was distributed within individual trees, among trees and among populations, we performed variance component analyses. Two analyses were performed; one for the response variable fruit size and one for seed size. Both analyses included population and tree as random factors.

In order to analyze the effect of the phenotypic selection exerted by birds and other factors affecting phenotypic trait differences among populations of *Crataegus monogyna*, we designed four LMM, one for each phenotypic trait as the response variable (fruit size and its CV and seed size and its CV). We included the total linear selection (*S*) on fruit and seed size and on their subindividual variation as predictor variables, together with crop size, latitude and precipitation. Some of the covariates have been measured at tree level while others correspond to population level measures (latitude, precipitation and selective pressure). This was adequately addressed by providing correct degrees of freedom for the latter and by including population as a random nesting factor. Note that selection differentials were assessed within each population. Therefore, selection differentials are related by nature to the differences among individual trees in each population but not to the phenotypic differences among populations. Thus, it is possible to include in the models the selection differentials (S) as predictors of the population’s phenotypes. When fruit size (average or variation) was the response variable, seed size variables (average and variation) were also included as predictor covariates.

However, average and variation of seed size were also analyzed as response variables. Stepwise backward elimination was used to progressively remove one variable at the time from the model until the *p*-value of any coefficient no longer exceeded 0.25 [24]. The statistical analyses were performed with SPSS for windows version 16.0 (SPSS Inc. version 16.1., Chicago, Illinois).

## Results

The variance components analysis for fruit size showed that 20.3% of the variance was distributed among populations whereas 44.8% was distributed among trees within the populations and 34.9% of the variance occurred within-individual trees. For seed size only 12.5% of variance was distributed among populations whereas 46.9% occurred among trees and 40.6% within trees (Figure S1).

We found that correlated traits, abiotic environment, geographic factors and selective pressures exerted by seed-dispersing birds explained part of the variation in the phenotypic traits under study.

**Figure 2:**
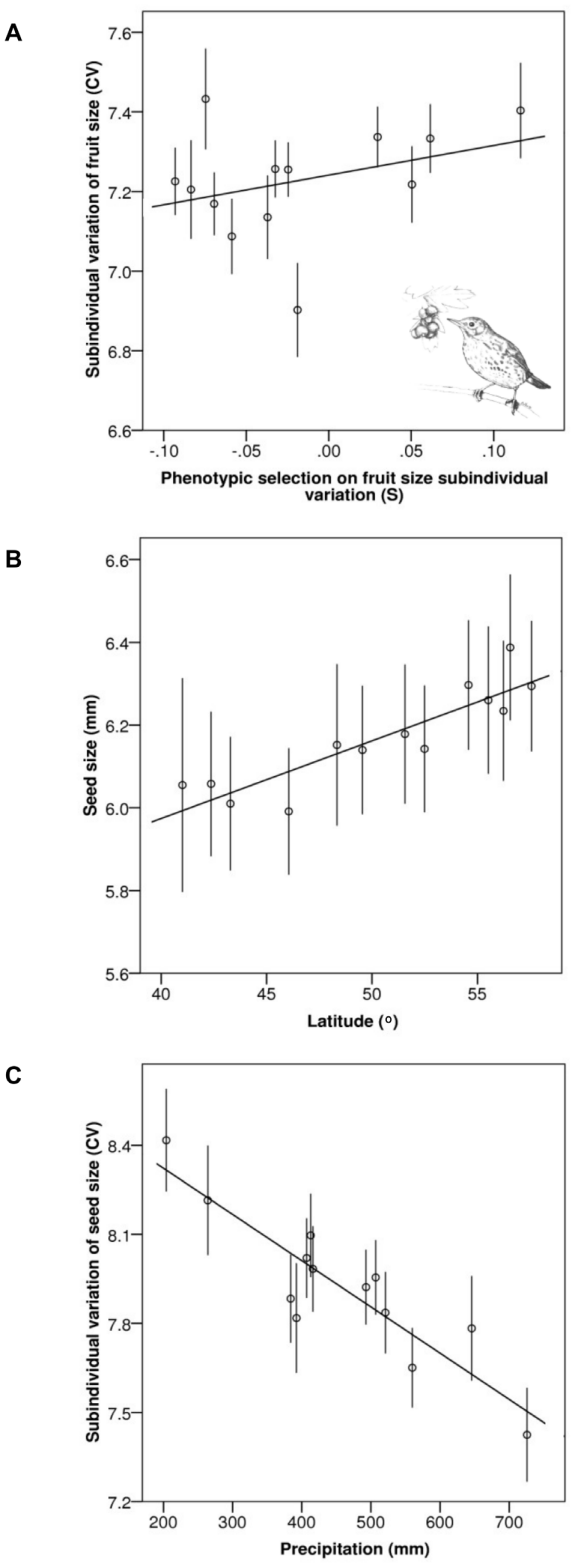
Differences among populations in phenotypic traits and their ecological correlates. Results of the LMM models fitted to the relationship between phenotypic traits and several population level covariates. (A) Relationship among phenotypic selective pressures exerted by seed dispersing birds and the subindividual variation of fruit size (P = 0.032). Note that the selective pressures are expressed as the selection differential exerted by the seed-dispersing birds (*S*). (B) Relationship among latitude and seed size (P = 0.017). (C) Relationship among precipitation and subindividual variation of seed size (P = 0.015). Dots represent estimated population-level means and bars show their standard errors.

Selective pressures exerted by birds affected the subindividual variation of fruit size at the population level, as shown by the significant effect of the total selection differential (*S*) for fruit size variation on this trait. Trees belonging to populations in which birds exerted negative selective pressures on fruit size variation exhibited lower levels of fruit size variation, and trees in populations were these selective pressures were positive presented higher values of fruit size variation (Table 1, Fig. 2A). Fruit size variation was also directly affected by seed size variation. Trees presenting higher levels of variation in seed size also presented more variable fruits (Table 1).

**Table 1:** Results of the reduced models (see Methods for details) to analyze the factors affecting the differences among populations in (A) average fruit size, (B) average seed size, (C) subindividual fruit size variation, and (D) subindividual seed size variation.

Response variable	Effect	Estimate	s.e	df	F	P
(A) Average fruit size	Seed size	0.365	0.052	1; 237.05	48.783	0.000
	Seed size CV	−0.107	0.050	1; 233.53	4.584	0.033
(B) Average seed size	Latitude	0.381	0.099	1; 10.67	14.595	0.003
(C) Fruit size CV	Seed size CV	0.334	0.0526	1;230.69	40.280	0.000
	Precipitation	−0.113	0.0541	1;9.29	4.362	0.065
	Selection coefficient (S) on fruit size CV	0.132	0.0518	1;8.54	6.525	0.032
(D) Seed size CV	Selection coefficient (S) on seed size CV	−0.127	0.769	1; 10.24	2.745	0.128
	Precipitation	−0.244	0.084	1; 10.69	8.467	0.015

Explanatory variables analyzed were seed size, precipitation, latitude and phenotypic selection (total selection coefficient (*S*) exerted by seed dispersers). Note that seed size was included as explanatory variable only when the response variable was average fruit size or fruit size subindividual variation.

Average fruit size was explained by correlated traits, specifically by, average seed size and seed size variation. Trees with higher variation in seed size exhibited smaller average fruit size and trees with larger seeds produced larger fruits (Table 1).

Average seed size and seed size variation were in turn determined by geographical and abiotic variables. Latitude was the only one of the variables to significantly determine differences among populations in average seed size of trees, which was larger for the trees belonging to more northerly populations (Table 1, Fig. 2B).

Seed size subindividual variation was explained by the total amount of precipitation in the population during the period of fruit and seed formation. Seed size was less variable within trees from populations with higher precipitation (Table 1, Fig. 2C).

## Discussion

The results of this research, despite admittedly correlative, show that differences in the subindividual variation of fruit size among different hawthorn populations throughout its latitudinal range of distribution were partly explained by the selective pressures exerted by seed-dispersing birds at each population. Interactions among species differ among populations due to, among other factors, differences in the community where these interactions occur. These differences form a geographic mosaic in which the evolution of one species is the result of selective pressures exerted locally by the rest of the species, as well as the gene flow among populations [25]; The greater is the distance among populations, the lower might be expected to be the gene flow and the higher the variation in the biotic and abiotic environments. Therefore, the phenotypic variation among distant populations may be the result of local adaptation, provided, of course, that these phenotypic traits are heritable. Heritability of within-individual variation of several traits has been documented [6], [7], [8], [9], [10].

Hawthorn fruits are dispersed in Europe almost exclusively by redwings, blackbirds and song thrushes [14]. Different selection regimes, throughout the latitudinal gradient in which hawthorn and its dispersers interact, may be attributed to several factors. First, the blackbird has a larger size and a less migrant behaviour than other thrush species and its contribution to total seed dispersal rate of hawthorn is higher towards the South [14]. Second, the species may differ as to the kind of microhabitats they visit after fruit consumption, which may affect the processes of seed predation, seed germination and establishment of new individuals [26]. Third, the differences in the social behaviour of the birds between migratory passing and wintering may also have an effect on their feeding preferences and, as a consequence, on the selective pressures exerted on plants. Alternatively, phenotypic variation found among populations could also be caused by selective pressures different from those measured here, as those exerted during other periods of the life cycle of the plants, either by seed predators [27], by the seed environment during germination [28] or by plant conditions during growth and survival [29]. We have studied the selective pressures exerted by seed predation, environment during germination and seedling growth [30] and we found selective pressures on within-individual variation of seed size in those post-dispersal stages which act in the same direction and with higher intensity than what had been reported for seed dispersers in the same hawthorn population [11]. The phenotypic differences in fruit size could also be explained by the effects caused by pre-disperser pulp and seed predators and by pathogens whenever they have effects related to fruit size. Nevertheless, predispersal seed predators as granivorous birds or mammals were not observed predating seeds and previous data suggest it is unlikely that the effects of pre-dispersal seed predators are important in this system [14].

The mutualistic interaction of ﬂeshy-fruit bearing plants with their dispersers is expected to have evolutionary consequences. Among the fruit characters susceptible to selection, fruit size is one of the traits most commonly reported to be selected by birds [11], [13], [31], and high levels of heritability for fruit size have been found [31]. Moreover, due to the close relationship between fruit and seed size within species [11], [32] and the variable effect of the latter in survival, germination and plant growth [27], [28], [29], [30], fruit size can be expected to affect plant reproductive success. Studies on fruit size selection by seed dispersers and its evolutionary consequences have, however, implicitly assumed that the selective pressures act directionally on the mean values of different size traits. In contrast to this approach, our results suggest that also subindividual variation in fruit and seed size is exposed to selective pressures. The possibility that selection may be exerted also on the skewness and on kurtosis of the distributions of phenotypic trait values within an individual, as well as on the spatio-temporal patterns of organization of this variation [1] remains to be explored.

Subindividual variation can be modified by selection, but it is a consequence of multiple factors such as developmental instability [4], architectural effects [2], [3], allocation processes [33], organ level reaction norms [1] and environmentally induced epigenetic effects [34]. The rarity of genetic mosaicism (the existence of several genetically distinct types of tissue within a single individual) in wild plants suggests that this is a negligible cause of subindividual variation [1]. Here we found that trees belonging to populations which received higher precipitation exhibit less variable seeds. Moreover precipitation was not correlated with crop size (data not shown). This would suggest that an important part of within-individual variation is due to competition for the resources among seeds in the same individual [35]. The scarcity of resources could also increase developmental instability. The relationship between resource availability and the within-individual variation in plant traits deserves further attention in future studies.

One of the most important factors affecting differences among populations in fruit sizes was seed size, which in turn was found to be related to latitude. The relationship of seed size and latitude had already been documented within species [36]. The geographic patterns related to variation in seed size suggest that the climatic characteristics which vary systematically with latitude (e.g. temperature, solar radiation) may play a role in the geographic variation of seed mass. Yet, there are other factors which may vary geographically and influence seed size, for instance, the availability of nutrients or the interactions with seed predators, both pre- and post-dispersal.

Demonstrating that a certain level of phenotypic variation is advantageous to individual plants [11] is only the first step in the task of demonstrating the adaptive nature of such variation. Showing that levels of subindividual variation vary among populations of a species and that this variation is explained by local selective pressures increases our understanding of the adaptive nature of within-individual variation.

The results of the present investigation, although of a correlative nature, are consistent with the emerging idea that selection exerted by animals affects subindividual variation in plants [1]. Phenotypic variance is composed of both, environmental and genetic variance, but subindividual variation originates from micro-environmental variation within a single genotype. Thus, selection on subindividual variation exerted by animals has the potential to alter the relative influence of the genetic and environmental components on plant phenotypic variance [1]. Subindividual variation seems to be an important component of biodiversity which may have important evolutionary implications yet to be understood.

## Supporting Information

Figure S1 **Variation in fruit (A) and seed (B) size per tree at each of the study populations.** Dots show the mean fruit or seed size per tree and bars indicate their standard deviation. Subindividual variation was assessed as the within-tree CV (s.d./mean). Therefore, this figure shows how the subindividual variation in fruit and seed size was distributed among trees and populations. Note that the populations are presented following its latitudinal order from South to North.
(TIF)
